# On the feasibility of *hearing* electrons in a 1D device through emitted phonons

**DOI:** 10.1038/s41598-021-85059-y

**Published:** 2021-03-09

**Authors:** Amit Verma, Reza Nekovei, Zahed Kauser

**Affiliations:** 1 Department of Electrical Engineering and Computer Science, Texas A&M University-Kingsville, Kingsville, USA; 2 Department of Electrical and Computer Engineering, University of Minnesota, Minneapolis, USA; 3 Present Address: Intel Corporation, Santa Clara, USA

**Keywords:** Nanoscience and technology, Nanoscale devices, Nanoscale materials, Materials science, Nanoscale materials, Theory and computation

## Abstract

This work investigates the vibrational power that may potentially be delivered by electron-emitted phonons at the terminals of a device with a 1D material as the active channel. Electrons in a 1D material traversing a device excite phase-limited acoustic and optical phonon modes as they undergo streaming motion. At ultra-low temperature (4 K in this study, for example), in the near absence of background phonon activity, the emitted traveling phonons may potentially be collected at the terminals before they decay. Detecting those phonons is akin to hearing electrons within the device. Results here show that traveling acoustic phonons can deliver up to a fraction of a nW of vibrational power at the terminals, which is within the sensitivity range of modern instruments. The total vibrational power from traveling optical and acoustic phonons is found to be in order of nW. In this work, Ensemble Monte Carlo (EMC) simulations are used to model the behavior of a gate-all-around (GAA) field-effect transistor (FET), with a single-wall semiconducting carbon nanotube (SWCNT) as the active channel, and a free-hanging SWCNT between two contacts. Electronic band structure of the SWCNT is calculated within the framework of a tight-binding (TB) model. The principal scattering mechanisms are due to electron–phonon interactions using 1st order perturbation theory. A continuum model is used to determine the longitudinal acoustic (LA) and optical (LO) phonons, and a single lowest radial breathing mode (RBM) phonon is considered.

## Introduction

Electrons passing through a device will spontaneously emit phonons with a high probability when their energy exceeds the threshold energy associated with the phonons. In bulk devices, or devices involving 2D materials, electrons have three or two degrees of freedom of motion, respectively. The emitted phonons, by extension, through the principles of conservation of energy and momentum, will also have multiple degrees of freedom. Therefore, the resulting crystal vibrations are spread over a 3D volume or 2D plane, respectively. In 1D or pseudo-1D materials, on the other hand, electrons can move only forward or back, say along positive x-axis or negative x direction, respectively, corresponding to the axis of the 1D material. If an electron moving along the x-axis (forward or back) emits a high energy optical phonon, for example, conservation of momentum dictates that the resulting phonon will also have a crystal momentum along the x-axis—a phase-space restricted scattering event—through either forward or back scattering event depending on the respective scattering probabilities. The electron, on the other hand, will again gain energy from the applied electric field, until it emits another phonon, and the process continues. This is known as the streaming-motion of charge-carriers^1^, and has been predicted for nanotubes and nanowires^2,3^. Depending on the background scattering activity, the process may continue over relatively very long material lengths until scattering events can result in a constant average charge-carrier drift-velocity.

An electron in a 1D material, therefore, may have multiple opportunities to emit phonons before being collected at a terminal, which all have crystal momentum in the same direction as the electron. Under room-temperature conditions these phonons may decay with a high rate^4^ (and more recently in Ref.^5^. for graphite, which is not a 1D material, but illustrates the point) or be undetectable over the background thermal noise. However, under ultra-low temperatures, say 4 K, there are essentially no background phonons, except for a few very low-energy acoustic phonons. It is mostly *quiet* at very low temperatures. In this case, charge-carrier transport within the 1D material will essentially be pseudo-transient, as also discussed in Ref.^6^, with steady-state conditions imposed at the contacts. Most of the phonons within the device will be due to phonon emission processes from charge-carriers. The detection of these emitted phonons will then depend on whether the power delivered by these phonons at the contacts is within the sensitivity range of current possible instrumentation setups.

## Methods

In order to quantify this, an EMC simulations-based device model is developed to model the electronic response of a (49,0) CNT as the active channel within two device configurations—a GAA FET, and as a free-hanging resistor—at an ultra-low temperature of 4 K. This is a semiconducting zigzag CNT, which has shown very pronounced streaming-motion even at room temperature. Following the details provided in Refs.^2,7^, TB calculations are used to calculate the band structure, and the lowest 7 subbands (14 with degeneracy) are included in the simulations. It should be noted that the total number of phonon dispersion branches in a CNT is given by *6 N*, where *N* is the number of hexagons within a CNT unit cell. However, not all the phonon branches that result from the detailed calculations are needed to model the possible electron–phonon interactions that may realistically occur. In most practical applied voltage (or electric field) cases, electrons will be confined to relatively lower energy subbands, and relatively few phonon dispersion branches need be considered for inter and intra-subband scattering within those subbands. Following Ref.^2^, graphene LA and LO phonons are zone-folded to obtain the corresponding phonon dispersion for the (49,0) CNT assuming free-standing boundary conditions. From the dispersion, 14 LA and 14 LO phonon branches are included in this work based on electron–phonon scattering selection rules. These phonon branches result in intra- and inter-subband electron scatterings within the included subbands. The lowest RBM phonon dispersion is also included, which causes intra-subband electron scattering. Scattering rates are computed using Fermi’s Golden Rule with deformation potential approximation. Within the simulation, for the calculation of the electron scattering rates, the electron band structure is divided into 18,000 grid points covering the 1st and 2nd Brillouin Zone (BZ), while the phonon dispersion is divided into 10,000 grid points covering the 1st BZ. Scattering rates calculations also include Umklapp processes. The computed band structure and scattering rates, and the final states after scattering are tabulated and utilized within the EMC simulations.

**Figure 1:**
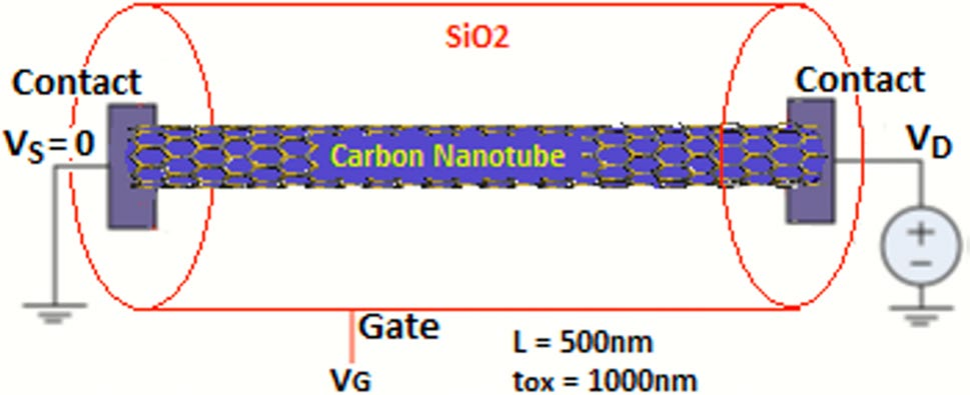
A schematic of the modeled gate-all-around (GAA) device. The work function difference between the ideal gate metal and the CNT is assumed to be zero.

The GAA device includes a 500 nm long (49,0) CNT, surrounded by a SiO_2_ layer with 1 µm radius, which in turn is surrounded by an ideal metal with no work function difference with the CNT. The CNT is assumed to be Ohmically contacted by ideal metals at both ends for both device configurations. Figure 1 depicts a schematic representation of the setup. Device electrostatics through the Poisson solver is implemented in integral form through Gauss’ Law^8^, and is calculated for each electron within the device every 0.5 fs. This time-step provided the highest numerical accuracy within the limitations of computational resources. The Monte Carlo solver is coupled to the electrostatic solver to obtain a self-consistent electrostatic potential profile. The electrostatic potential felt by each electron is the sum of the contributions from all other electrons as well as the voltages at the contacts, i.e., each electron in the CNT feels the electrostatic potential due to all other electrons and the electrodes. For simplicity of implementation the CNT is treated as dynamically one-dimensional. The CNT is divided uniformly along its length with a grid size of 2.5 Å. Therefore, every 0.5 fs, a matrix of 2000 × 2000 is created for each electron within the device to determine the electrostatics that determines its motion. Most simulations were carried out for 2 ns. In addition to electrons, both acoustic and optical phonons are also tracked as they are emitted after every scattering process. The energy ($\hbar \omega )$ and crystal momentum ($\hbar k)$ of each emitted phonon corresponds to the energy and crystal momentum lost by an electron in the scattering processes. The group velocity of the phonon, *dω/dk*, is determined from the tabulated phonon dispersion. Most CNT phonon lifetime results reported are for higher temperatures. At 300 K, optical phonon reported lifetime is approximately 1 ps^4^, and goes down as *1/T* as temperature increases. This relationship will certainly not hold at 4 K, and phonon lifetimes may be significantly longer than 0.1 ns here, following the same law, because of the near-absence of any mechanisms to provide any significant damping on phonon transport. We conducted our simulations for a phonon lifetime of 0.1 ns and an arbitrary long lifetime of 1 s, and assumed equal lifetimes for acoustic and optical phonons. If a phonon is deemed to have dissipated within our simulation, its energy and crystal momentum are assumed to be instantaneously lost, and are not accounted for in the final tally of total phonon energy delivered to the contacts of the device. This is a worst-case scenario. What is likely to occur is a slow decay of phonon energy and crystal momentum, resulting in smaller energy phonons, carrying energy and crystal momentum dictated by the laws of conservation. It is also assumed that background phonon activity in the surrounding media (contacts, insulator) at this low temperature will also be mostly absent, and therefore will have negligible influence on electron and phonon transport within the CNT for the CNT length considered. It is seen that within a 500 nm CNT length, a 10-order magnitude difference in phonon lifetime—0.1 ns to 1 s—results in only a relatively small difference in phonon power delivered to the contacts, which further suggests that the surrounding environment may not have a significant influence on phonon transport within a 500 nm length of this CNT.

At *t* = *0* s, the voltages are assumed turned on. Before this, the CNT is assumed devoid of free charges, which is reasonable given the low intrinsic concentrations of these materials, even at room temperatures^9^. Electrons are injected into the device from the source and collected at the drain. Relative permittivity within the CNT is considered ε_r_ = 3.9, corresponding to the surrounding SiO_2_ permittivity for the GAA FET setup, and ε_r_ = 1.0, for the free-hanging CNT.

## Results and discussions

**Figure 2:**
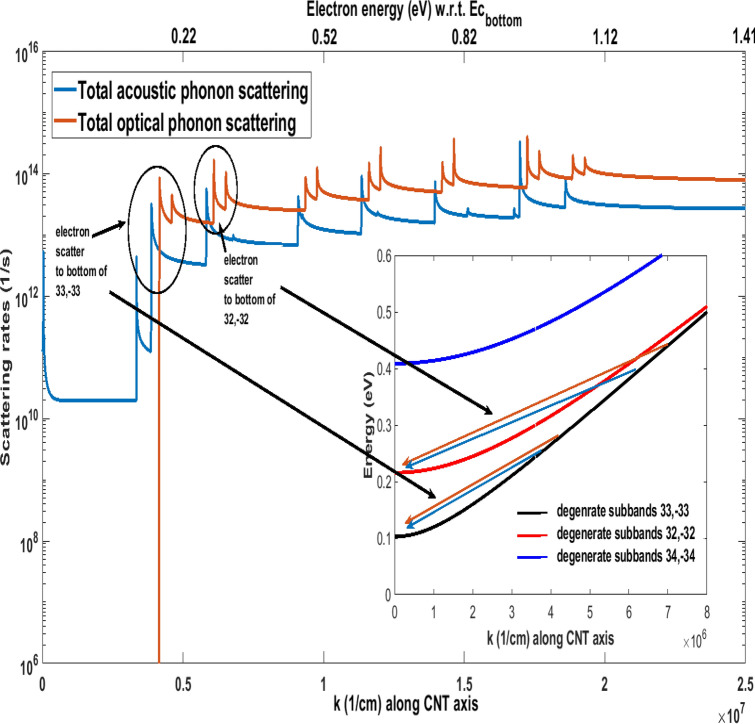
Total electron-optical and electron-acoustic phonon scattering rates for the (49,0) CNT at 4 K for the lowest two degenerate subbands with quantum numbers 33 and − 33 (representing discrete values of allowed wave vectors in the circumferential directions). The lowest three (six with degeneracy) subbands are partially depicted in the inset. The scattering rates are dominated by emission processes, except for the first peak near Γ point, which corresponds to acoustic phonon absorption scattering. Scattering peaks correspond to electron transitions to bottom of subbands, and play a significant role in defining electron transport. The first two sets of emission peaks are highlighted in circles and the corresponding electron transitions are illustrated by arrows in the inset.

Electron–phonon scattering rates calculations at 4 K in this work show that most absorption processes are non-existent. The only exception is acoustic phonon absorption scattering from the lowest phonon branch at low phonon energies. This corresponds to an intra-subband electron transition from near the bottom of a subband to a state also near that subband bottom. This particular scattering rate falls rapidly with increasing crystal momentum, *k*, and becomes negligible as electron energy increases. Figure 2 shows the total acoustic and optical phonon scattering rates for the lowest conduction subband. All peaks in the scattering rates, except for the acoustic phonon scattering peak around the Γ point, correspond to the onset of phonon emission, and a simultaneous inter or intra subband electron transition. The particular first acoustic phonon peak near the Γ point corresponds to phonon absorption with an intra-subband electron transition.

**Figure 3:**
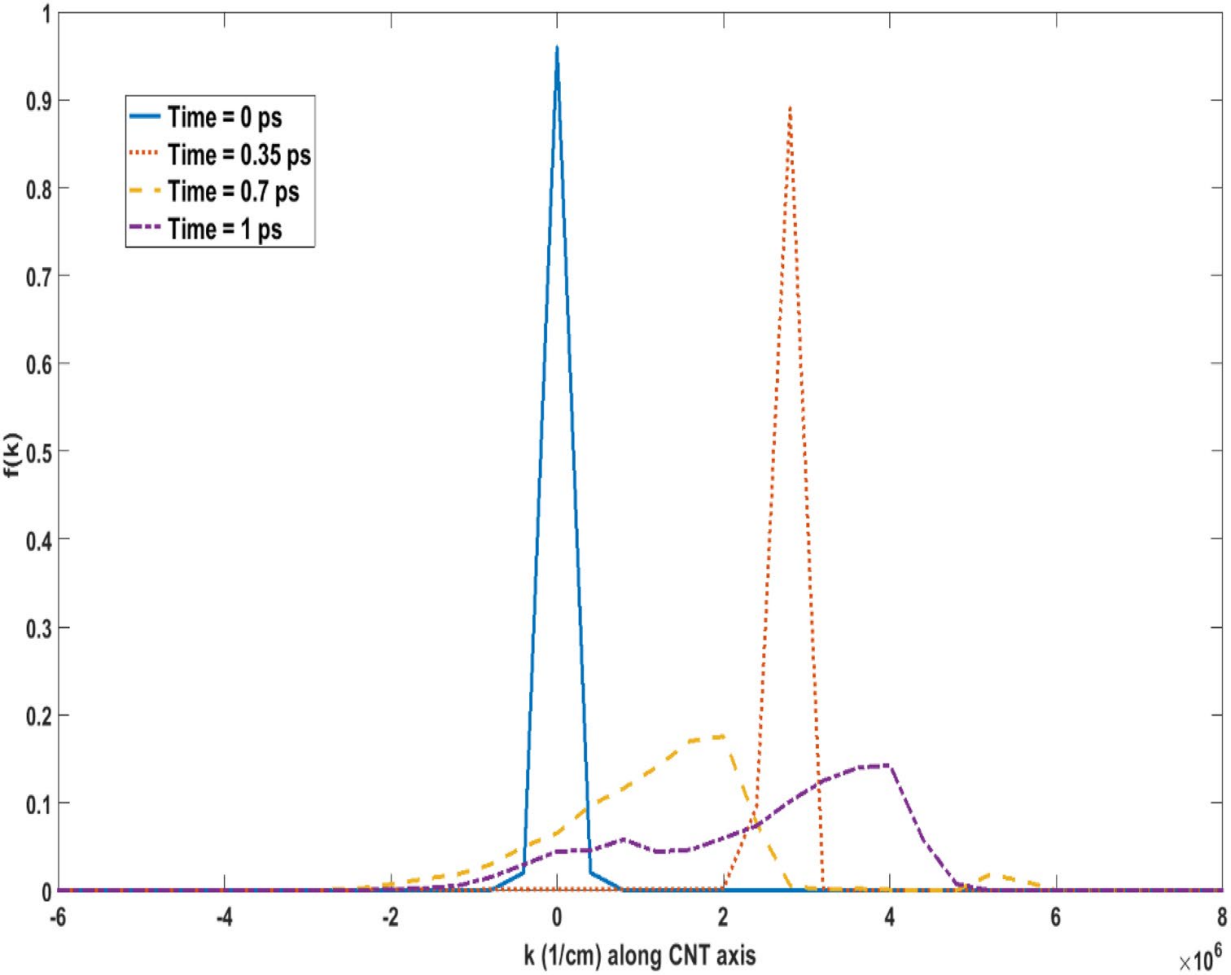
Transient time evolution of the electron distribution function for a 5 kV/cm electric field at 4 K for (49,0) CNT. The electron distribution is seen to be significantly affected by the first set of emission scattering peaks around k = 4 × 10^6^/cm seen in Fig. 2. Please note that the distribution at *t* = *0* is a thermal equilibrium Gaussian distribution that appears as a sharp peak in the *k*-axis scale used.

**Figure 4:**
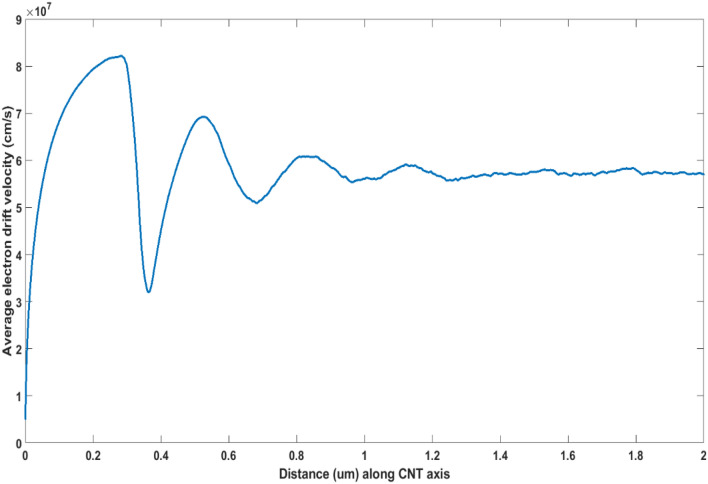
Average electron drift velocity at 5 kV/cm and 4 K versus distance along CNT axis. The velocity oscillations, caused by phonon emission processes, can be seen to be significant over long lengths.

Before investigating the device responses, electron transport within an ideal, infinitely long (49,0) CNT, guided by the above calculated scattering rates and axially-oriented uniform electric field was characterized. Figure 3 shows the transient time evolution of the distribution of an electron gas within the CNT when a uniform electric field of 5 kV/cm is applied at *t* = *0* ps. The electron gas was initially (time *t* = *0*) at thermal equilibrium at 4 K, with an equilibrium thermal Gaussian distribution within the first subband. It should be noted that the sharp peak in the distribution seen in Fig. 3 at *t* = *0* is primarily the result of *k*-axis scale utilized to depict the time-evolution of the distribution. The role of the first set of emission peaks near $k = 4 \times 10^{6} \,\,{\text{cm}}^{ - 1}$ in Fig. 2 in influencing the motion of electrons can be clearly gauged from Fig. 3. Here, the electron gas is seen to be “bouncing” back-and-forth as electrons lose crystal momentum to scattering and gain it from the electric field. The entire electron distribution undergoes a near ballistic transport with relatively insignificant influence by acoustic phonon absorption till approximately the first *t* = 0.35 ps, when the distribution meets with the first set of phonon emission scattering peaks around $k = 4 \times 10^{6} \,\,{\text{cm}}^{ - 1}$ seen in Fig. 2. At this point, significant number of electrons lose crystal momentum, and undergo transition to the bottom of the subband. Thereafter, the electrons again undergo acceleration under the influence of the applied electric field, and the process repeats while the distribution broadens as electrons occupy broader *k*-values through different scattering mechanisms. This behavior, or the streaming motion, can persist for long distances before diffusive transport becomes dominant. This can be seen in Fig. 4, which shows the oscillations in electron average drift velocity persisting for over a µm at 5 kV/cm. It is important to note that even when velocity oscillations disappear, electrons still undergo phonon scattering mostly through emission processes, because phonon absorption scattering is negligible at 4 K. A single electron can therefore cover a relatively large distance undergoing acceleration before it scatters.

Within the above described devices, depending on the applied voltages, steady-state current values at the drain contact are reached within a few picoseconds. Thereafter, for the GAA FET, for a phonon lifetime of 0.1 ns, a gate voltage of 0.5 V, and source-drain voltage of 1 V, we find that approximately 0.25 nW of optical phonon power is collected at the drain, while 0.32 nW of acoustic phonon power is collected at the drain. This corresponds to a total of approximately 0.6 nW of total vibrational (phonon) power at the drain. A much smaller (~ 15 times smaller) phonon power is collected at the source terminal. Concomitantly, a current of approximately 0.47 µAmp flows into the drain. For an arbitrarily long phonon lifetime of 1 s, the acoustic phonon power collected at the drain terminal increases slightly to approximately 0.35 nW. With the same large phonon lifetimes of 1 s, but with a gate voltage of 0.75 V and source-drain voltage of 1.5 V, approximately 0.7 nW of acoustic phonon power and 1 nW of optical phonon power is delivered to the drain terminal. Approximately 0.17 nW of acoustic phonon power is also delivered to the source terminal, and optical phonon power is approximately 1/10th of that.

The advantage of a gated device here is in being able to obtain measurable phonon power and currents with reasonable contact voltages. In the case of the free-hanging CNT, an acoustic phonon power of approximately 0.9 nW and optical phonon power of approximately 0.9 nW are delivered at the drain for a relatively high applied voltage of 4 V between the two contacts.

In conclusion, this work investigated whether phase-limited phonon emission activity resulting from the streaming motion of electrons in 1D materials may potentially be high enough to be detected at the terminals of a device (akin to *hearing* those electrons as they traverse the device) when the background phonon activity is very low to absent, such as at ultra-low temperatures. The resultant values of total phonon power delivered to the device terminals in this work, for a single (49,0) CNT, are seen to be on the order of a nW. Acoustic phonon power delivered is less than a nW. This phonon power potentially lies within the range of the sensitivity of a state-of-the-art Atomic Force Microscope (AFM)^10^ (but not precluding other current or emerging instrumentation setup), making these vibrations possibly detectable dependent upon the nature of their coupling to external instrumentation setup contacting the device and challenges associated with extreme low temperature measurements. AFM probing of CNTs and other 1D materials at elevated temperatures has been demonstrated in several works over the years (see, for example, Ref.^11^). Specific to this work, higher voltage requirements for reasonable phonon power notwithstanding, a free-standing CNT may be a relatively easier device setup than a GAA-FET. The applied voltage requirement can be reduced by utilizing a backgated structure. In both these cases (free-standing and backgated device structures), the CNT can be directly probed at any point on its surface to detect phonon vibrations, which reduces fabrication and setup complexity. In the GAA-FET structure, the AFM probes can only be at the contacts, in addition to relatively more complicated fabrication requirements. Other methods of detecting electron-emitted phonons may also be considered, such as optical probes utilized for the detection of coherent phonons in Ref.^12^. The required sensitivity for such setups, apropos detecting such phonons, is not obvious compared to AFMs^10^. However, optical probing is contactless, which is an advantage, particularly when the device needs to be cooled to ultra-low temperatures. It should be noted that both optical and acoustic phonons are emitted by electrons moving through the device and may be collected at the terminals. While this work looks at specific device setup, contact-type, and a single CNT, it may be more broadly applicable, as long as sufficient charge-carriers are injected into the device and undergo streaming motion. It may also be applicable to other nanomaterials, such as silicon nanowires, since they too have shown the potential for streaming motion^3^. The total power collected may also be increased by incorporating more nanotubes or nanowires within a device. Such directed phonons may potentially find applications in compositional studies of materials. It is also possible that they may be potential source of information transmitted through a device or circuit—a phonon-based circuit.

## Data Availability

The data supporting the findings of this study are available within the paper. The FORTRAN codes used to generate the data are available from the authors upon reasonable request subject to export control laws.
